# Mouse model resembling human lung cancer

**DOI:** 10.18632/oncotarget.1017

**Published:** 2013-05-02

**Authors:** Zuoxiang Xiao, Qun Jiang, Jami Willette-Brown, Yinling Hu

**Affiliations:** Laboratory of Experimental Immunology, Cancer and Inflammation Program, Center for Cancer Research, National Cancer Institute, Frederick Maryland; Laboratory of Experimental Immunology, Cancer and Inflammation Program, Center for Cancer Research, National Cancer Institute, Frederick Maryland; Laboratory of Experimental Immunology, Cancer and Inflammation Program, Center for Cancer Research, National Cancer Institute, Frederick Maryland; Laboratory of Experimental Immunology, Cancer and Inflammation Program, Center for Cancer Research, National Cancer Institute, Frederick Maryland

**Figure 1:**
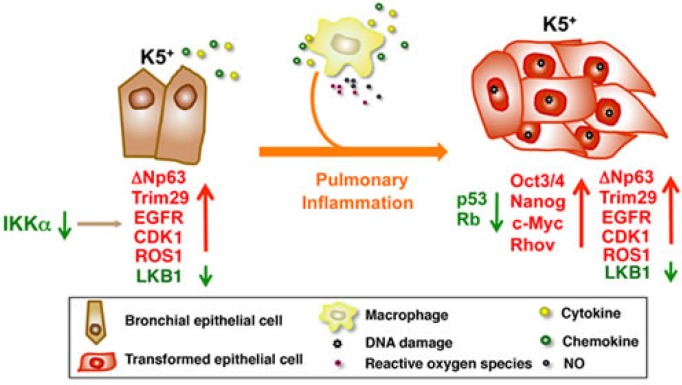
IKKα downregulation dysregulates the expression of multiple oncogenes and tumor suppressors in K5+ lung epithelial cells The mutant macrophages increase inflammatory responses and oxidative stress to promote DNA damage in IKKα-mutant K5+ lung epithelial cells, which further dysregulate the levels of multiple oncogenes, tumor suppressors, and stem cell genes, thereby promoting the IKKαlowK5+p63hi cell transition to tumor cells in L-IkkαKA/KA lungs. Arrow down (green), downregulation; arrow up (red), upregulation; crossing two lines, inhibition.

Following our previous findings that IKKα reduction promotes carcinogen-induced skin carcinogenesis, somatic IKKα deletion induces spontaneous skin papillomas and squamous cell carcinomas (SCCs) in *Ikkα*-floxed mice, and overexpressed IKKα represses carcinogen-induced skin carcinogenesis [1-3], we recently reported that kinase-dead *Ikkα* knockin (*Ikkα*^*KA/KA*^) mice develop spontaneous lung, forestomach, and skin SCCs, which are associated with IKKα downregulation and inflammation [4]. We further studied the pathogenesis of lung SCCs after stabilizing the skin condition by introducing the transgenic Lori.IKKα into the skin of *Ikkα*^*KA/KA*^ (*L*-*Ikkα*^*KA/KA*^) mice (Figure 1).

The human *Ikkα* gene is located at 10q24.31, close to *Pten* (10q23). Many studies have suggested that alterations within the 10q22-10q26 region may be involved in human SCC development. IKKα downregulation and *Ikkα* mutations and deletions have been reported in various types of human SCCs. A point mutation-mediated IKKα deletion has been identified in a lethal human syndrome, in which multiple organs are malformed [5]. Chemical carcinogen treatment induces *Ikkα* mutations and deletions in mouse skin SCCs [1]. These findings underscore the importance of IKKα in the pathogenesis of these human diseases.

Lung cancer is the leading cause of cancer mortality worldwide. Lung SCC, one of major types of lung cancers, is strongly associated with smoking that contains carcinogens. Smoking can also act as an inflammatory irritator. It is thought that SCCs, frequently found in the upper and central regions of the lungs, are derived from the basal cells (a type of squamous epithelial cell) of the pseudostratified epithelial layer of bronchi; whereas adenocarcinomas (ADCs) are derived from the epithelial cells of alveoli. Many molecular alterations, such as deregulated EGFR, PIK3CA, p53, Rb, and c-Myc, have been identified in human lung SCCs, but mice overexpressing or lacking these genes do not well recapitulate the development of lung SCCs, although a proportion of *K-ras*^*G12D*^;*Lkb1*^*−/−*^ mice develop mixed SCCs and ADCs in the lungs [4,6].

Biomarkers of increased p63, Trim29, and keratin 5 (K5) are used to distinguish human lung SCCs from ADCs. We also have demonstrated increased p63, Trim29, and K5 levels, deregulated RhoV, CDK1, IGF, ROS1, c-Myc, p53, Rb, and LKB1 expression, and elevated EGFR, ERK, and p38 activity in lung SCCs derived from *L*-*Ikkα*^*KA/KA*^ mice [4]. Therefore, the mouse lung SCCs resemble human cancers. Interestingly, many human lung SCCs express elevated Sox2; however, the mouse lung SCCs express elevated Nanog and Oct3/4, but not Sox2 (our unpublished data), suggesting that human and mouse lung SCCs may express a different spectrum of stem cell regulators [4]. The nuclear IKKα has been shown to regulate the expression of many genes in keratinocytes through an epigenetic mechanism [2]. We further revealed that IKKα downregulation upregulates the level of H3K4me3, a positive transcription mark, and reduces the level of H3K27me3, a negative transcription mark, on the loci of *p63* and *Trim29* in the *Ikkα*^*KA/KA*^ SCC cell line and *Ikkα*^*KA/KA*^ MEFs compared to wild-type cells. Reintroducing IKKα reverses the levels of H3K4me3 and H3K27me3, represses the expression of ΔNp63 and Trim29 in *Ikkα*^*KA/KA*^ cells, and inhibits the proliferation of the *Ikkα*^*KA/KA*^ SCC cell line [4]. A similar regulatory mechanism for the expression of ΔNp63 and Trim29 was detected in the human lung SCC cell line. Thus, the IKKα-mediated epigenetic regulation plays a role in the pathogenesis of lung SCCs. In addition, the elevated ΔNp63 level, increased EGFR and ERK activity, and K5^+^ cell expansion have been detected in the epidermis of *Ikkα*^*f/f*^/K5.CreER and *Ikkα*^*−/−*^ mice (our unpublished result) and in the *L*-*Ikkα*^*KA/KA*^ lungs [4,7], suggesting that some similar molecular mechanisms may be involved in the pathogenesis of skin and lung SCCs in these different IKKα mutant mice.

Previously, we showed that IKKα inactivation interrupts early hematopoietic cell development, resulting in increased myeloid cells in *Ikkα*^*KA/KA*^ mice [8]. We further demonstrated that these increased macrophages are necessary for promoting lung SCC formation in *L*-*Ikkα*^*KA/KA*^ mice by increasing DNA damage to deregulate tumor suppressors, oncogenes, and stem cell regulators (Figure 1) [4]. We detected markedly increased macrophages, T cells, and multiple mediators in *L*-*Ikkα*^*KA/KA*^ lungs compared to wild-type lungs. NF-κB activity is increased in CD45^−^ cells isolated from *L*-*Ikkα*^*KA/KA*^ lungs as well as in a cell line derived from the *L*-*Ikkα*^*KA/KA*^ lung SCC [4]. The increased NF-κB activity may contribute to multiple elevated mediator products and anti-apoptotic activities in lung epithelial cells. Importantly, we detected increased macrophages, and reduced IKKα levels in human lung SCCs and their adjacent lung tissues, indicating that IKKα reduction may not only promote lung epithelial cell malignant transformation, but also enhance inflammatory responses.

We have identified similar multi-molecular alterations in mouse and human lung SCCs [4]. Our immediate goal is to identify specific molecules, cells, or pathways that can be targeted for therapy, which will benefit human cancer patients. Using this model, we will investigate conditions for lung SCC metastasis and essential pulmonary inflammatory cell development, as well as the relationship between lung SCC and ADC pathogenesis.
